# Preoperative hepatic encephalopathy predicts early postoperative complications after orthotopic liver transplantation: a retrospective cohort study

**DOI:** 10.1016/j.clinsp.2026.101176

**Published:** 2026-09-30

**Authors:** Imran Muhammad, Faisal UL Rehman, Jinzhen Cai

**Affiliations:** a Organ Transplantation Center, The Affiliated Hospital of Qingdao University, Qingdao, Shandong, China; b Precision Medicine Center of Oncology, The Affiliated Hospital of Qingdao University, Qingdao, Shandong, China

**Keywords:** Orthotopic liver transplantation, Hepatic encephalopathy, Postoperative complications, Logistic regression, Perioperative outcomes

## Abstract

**Background:**

Early postoperative complications remain an important source of morbidity after Orthotopic Liver Transplantation (OLT). Identifying preoperative factors associated with these complications may help improve perioperative risk assessment and postoperative management. This study evaluated clinical predictors of early postoperative complications following OLT, with particular emphasis on preoperative hepatic encephalopathy.

**Methods:**

We conducted a retrospective cohort study of adult patients who underwent OLT at the Organ Transplantation Center of The Affiliated Hospital of Qingdao University between January 2014 and December 2024. The primary endpoint was the occurrence of at least one clinically significant postoperative complication within 30 days after transplantation. Potential predictors were evaluated using univariate analyses followed by multivariable logistic regression. Model discrimination was assessed using receiver operating characteristic curve analysis.

**Results:**

Among 1,478 liver transplant recipients, 130 (8.8%) developed at least one early postoperative complication. On univariate analysis, body height, body weight, serum albumin, total bilirubin, international normalized ratio, intraoperative blood loss, hepatic encephalopathy, and ascites were associated with postoperative complications. In the multivariable model, preoperative hepatic encephalopathy remained independently associated with an increased risk of early postoperative complications (Odds Ratio [OR = 1.999]; 95% Confidence Interval [95% CI 1.23‒3.25]; p = 0.005). Greater intraoperative blood loss showed a borderline association with postoperative complications (OR = 1.00007; p = 0.052). The model demonstrated moderate discriminatory performance, with an area under the receiver operating characteristic curve of 0.699.

**Conclusions:**

Preoperative hepatic encephalopathy was independently associated with early postoperative complications after OLT. These findings suggest that hepatic encephalopathy may be useful as a readily available marker for identifying recipients at increased postoperative risk. Further prospective and multicenter studies incorporating comprehensive donor, recipient, and perioperative variables are warranted to validate these findings and improve postoperative risk prediction.

## Highlights

• Preoperative hepatic encephalopathy independently predicted early post-OLT complications.
• Hepatic encephalopathy was associated with an approximately twofold increase in complication risk.
• Lower body weight and greater intraoperative blood loss were linked to adverse outcomes.
• Analysis of 1,478 liver transplant recipients provides robust real-world evidence.
• Findings support improved perioperative risk stratification in liver transplantation.

## Introduction

Orthotopic Liver Transplantation (OLT) is widely accepted as the definitive treatment for end-stage liver disease and selected cases of acute liver failure. Over the past several decades, advances in surgical techniques, perioperative care, organ preservation, and immunosuppressive therapy have significantly improved postoperative survival rates following liver transplantation.^1^ Despite these improvements, postoperative complications remain a major challenge and continue to contribute to increased morbidity, prolonged hospitalization, graft dysfunction, and mortality after transplantation.^2^ Early postoperative complications after liver transplantation include vascular thrombosis, biliary complications, renal dysfunction, pleural effusion, infectious complications, and graft dysfunction.^3^^,^^4^ Therefore, identifying perioperative factors associated with postoperative complications is important for improving patient outcomes and optimizing perioperative management strategies.^5^ Recipients undergoing liver transplantation frequently present with severe hepatic dysfunction accompanied by metabolic abnormalities, malnutrition, coagulation disorders, portal hypertension, and impaired renal function.^6^ Several preoperative clinical indicators, including serum bilirubin, albumin, creatinine, International Normalized Ratio (INR), ascites, and hepatic encephalopathy, have been investigated as predictors of postoperative outcomes after liver transplantation.^7^ Recent studies have highlighted the increasing importance of perioperative risk assessment to identify transplant recipients at high risk of postoperative complications and to support individualized perioperative management. Despite advances in prediction models and perioperative care, there remains a need for simple and readily available clinical markers that can be incorporated into routine pretransplant evaluation.^8^, ^9^, ^10^

Hepatic encephalopathy is considered a manifestation of advanced liver disease and reflects impaired hepatic metabolic function and reduced physiological reserve.^11^ The pretransplant hepatic encephalopathy may be associated with poorer postoperative recovery and increased postoperative morbidity after liver transplantation. In addition, intraoperative factors such as excessive blood loss and prolonged operative complexity may further increase the risk of postoperative complications through hemodynamic instability, transfusion-related effects, and ischemia-reperfusion injury.^12^

In addition to neurological impairment, hepatic encephalopathy is closely associated with systemic inflammation, immune dysfunction, malnutrition, and sarcopenia, which may reduce physiological resilience during the perioperative period. These factors can impair recovery, increase vulnerability to infection and organ dysfunction, and contribute to adverse postoperative outcomes after liver transplantation. Based on these pathophysiological considerations, we hypothesized that preoperative hepatic encephalopathy may serve as a clinically relevant predictor of early postoperative complications following orthotopic liver transplantation.

## Methods

### Study design and patient selection

A retrospective observational study was performed using data obtained from adult patients who underwent Orthotopic Liver Transplantation (OLT) at the Organ Transplantation Center of The Affiliated Hospital of Qingdao University between January 2014 and December 2024. Clinical information was collected from the institutional transplantation database and electronic medical records. Eligible patients were adult recipients who underwent primary orthotopic liver transplantation and had complete perioperative clinical data and documented postoperative outcomes within 30-days after transplantation. Patients with incomplete perioperative information, missing postoperative outcome data, or those who underwent retransplantation were excluded from the analysis. Liver transplantation was performed for end stage liver disease, acute liver failure, hepatocellular carcinoma meeting transplant criteria, and other accepted indications according to institutional practice. Donor organs were procured from multiple external hospitals through the national organ allocation system. Consequently, complete donor-related information and several perioperative variables were not consistently available across the retrospective study cohort. Although some donor variables could be retrieved retrospectively, they were incomplete or inconsistently recorded over the study period and therefore were not incorporated into the multivariable analysis. This retrospective cohort study was reported in accordance with the Strengthening the Reporting of Observational Studies in Epidemiology (STROBE) guidelines. The study selection process and patient grouping are summarized in Fig. 1.

**Fig. 1:**
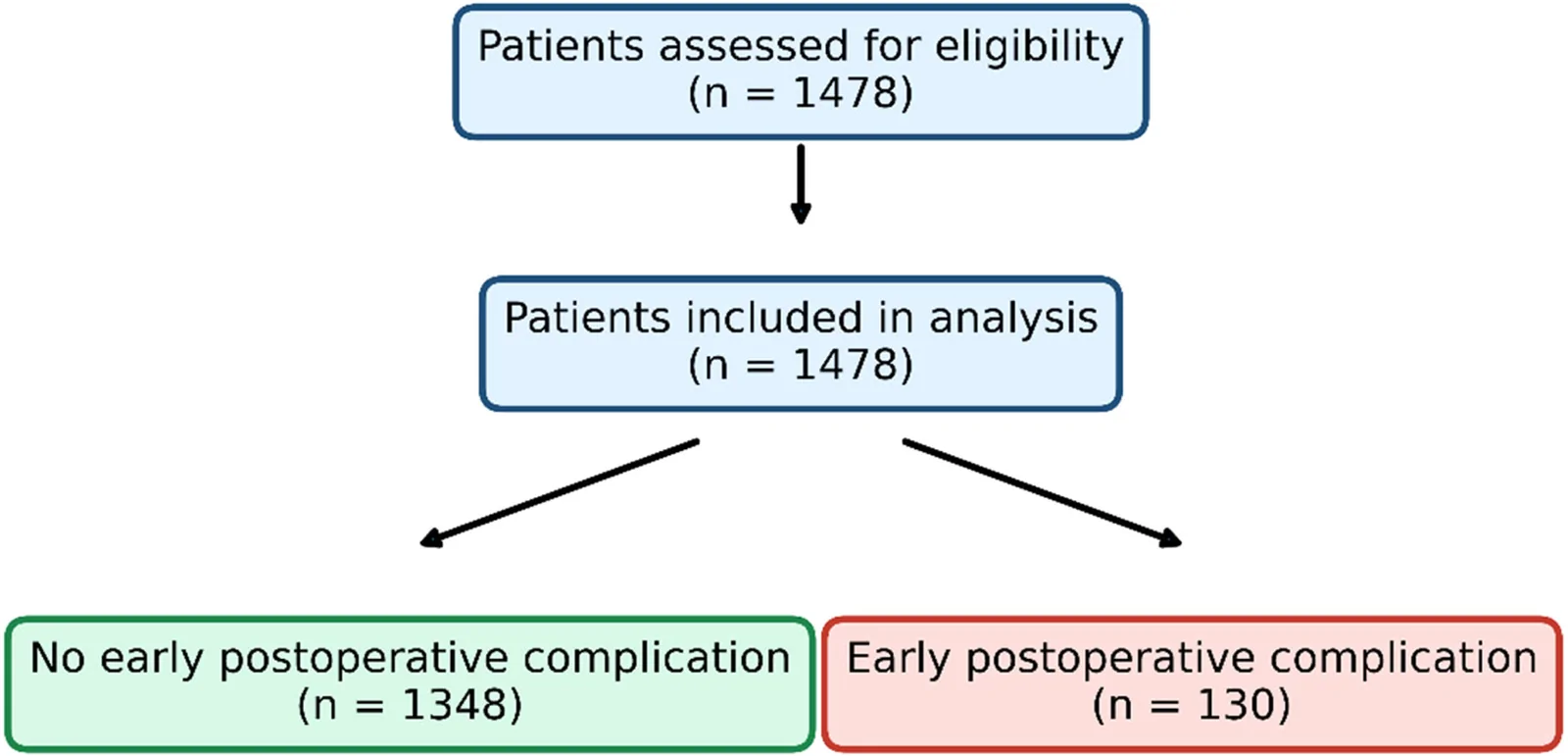
Study flowchart of patient selection and grouping.

### Clinical variables

Preoperative recipient variables included age, body height, body weight, hepatic encephalopathy, ascites, serum albumin, creatinine, total bilirubin, and International Normalized Ratio (INR). Preoperative hepatic encephalopathy was identified from the electronic medical records as a documented clinical diagnosis before transplantation and was analyzed as a binary variable (present or absent). Detailed information regarding the diagnostic criteria, West Haven grade, and whether hepatic encephalopathy represented a first or recurrent episode was not consistently available in the retrospective database and therefore could not be evaluated. Rifaximin was not routinely used in our study population, whereas lactulose was administered when clinically indicated in patients with hepatic encephalopathy. Preoperative ascites was identified from clinical documentation and radiologic evaluation recorded in the electronic medical records and were analyzed as a binary categorical variable (present versus absent). Intraoperative variables included estimated blood loss during transplantation.

Early postoperative complications were defined as the occurrence of at least one clinically significant adverse event within 30-days after orthotopic liver transplantation. The predefined complications included hepatic artery thrombosis, portal vein thrombosis, hepatic vein thrombosis, bile leakage, intra-abdominal bleeding, primary nonfunction, delayed graft function, pleural effusion, urinary tract infection, wound infection, renal failure, opportunistic infection, and cytomegalovirus infection. Each complication was identified from the electronic medical records based on routine clinical assessment by the transplant team, incorporating clinical findings, laboratory investigations, and imaging studies when appropriate. For the primary endpoint, all complications were considered equally, and recipients who developed one or more postoperative complications were classified into the complication group regardless of the total number of events experienced. Patients without any documented complication within 30-days after transplantation were classified into the non-complication group. The frequency of each individual complication is presented in Supplementary Table 1.

### Data processing

Original clinical data were recorded using Chinese-language variables and were translated into standardized English variables before statistical analysis. Variables with invalid or biologically implausible values were reviewed and corrected during the data-cleaning process. Only one creatinine value was missing and was replaced using median imputation. No missing values were identified for the remaining variables included in the analysis; therefore, no additional imputation procedures were required.

### Statistical analysis

Continuous variables were expressed as mean ± standard deviation or median with inter quartile range according to data distribution. Categorical variables were presented as frequencies and percentages. Normality of continuous variables was evaluated using the Shapiro-Wilk test. Since most continuous variables demonstrated non-normal distribution, comparisons between groups were performed using the Mann-Whitney *U* test. Categorical variables were compared using the Chi-Square test. Variables demonstrating a significance level of p < 0.10 in univariate analysis, together with clinically relevant variables consistently available in the study database that were considered for inclusion in the multi-variable logistic regression model to identify independent predictors of early postoperative complications. Although additional perioperative variables, including donor characteristics, were partially retrieved during manuscript revision, these data were not consistently available for the entire cohort and therefore were not incorporated into the original multi-variable model to preserve the consistency of the predefined analytical dataset. Several established perioperative risk factors, including donor characteristics, ischemia times, operative duration, blood transfusion, retransplantation status, recipient comorbidities, and MELD score, were not consistently available for the entire study cohort and therefore could not be included in the final regression model. In addition to statistical significance, clinically relevant perioperative variables associated with postoperative outcomes after liver transplantation were considered during model construction. Multi-variable logistic regression analysis was performed to minimize potential confounding effects and identify variables independently associated with postoperative complications. The available sample size, including 130 postoperative complication events among 1,478 recipients, was considered adequate for the multivariable logistic regression analysis performed. Correlation analysis was performed before model construction to evaluate potential multicollinearity among candidate variables included in the regression model. No clinically significant multicollinearity was identified among the candidate variables. Odds Ratios (ORs) and 95% Confidence Intervals (95% CIs) were calculated. The predictive performance of the final regression model was evaluated using Receiver Operating Characteristic (ROC) curve analysis, and the Area Under the Curve (AUC) was calculated. All statistical analyses were performed using Python in Google Colaboratory (Google LLC, Mountain View, CA, USA). A two-sided p-value < 0.05 was considered statistically significant.

## Results

### Patient characteristics

A total of 1,478 patients who underwent orthotopic liver transplantation were included in the final analysis. Among them, 130 patients (8.8%) developed early postoperative complications, while 1,348 patients (91.2%) had no postoperative complications during the early postoperative period. The most frequently observed complications were intra-abdominal bleeding, opportunistic infection, and pleural effusion. The distribution of individual postoperative complications is illustrated in Fig. 2.

**Fig. 2:**
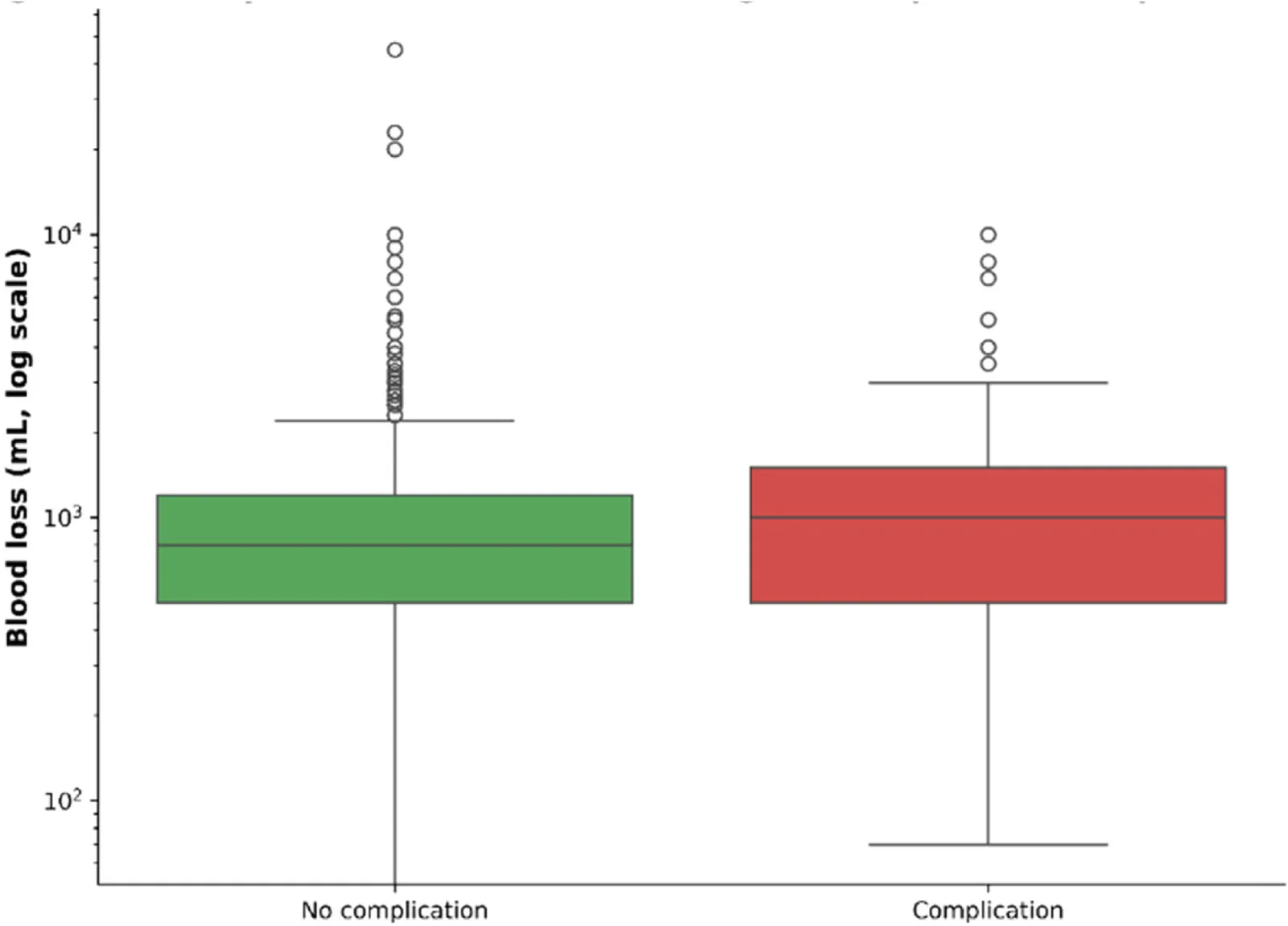
Distribution of individual early postoperative complications after orthotopic liver transplantation.

Baseline demographic and perioperative characteristics of the study population are summarized in Table 1.

**Table 1:** Baseline demographic and perioperative characteristics of patients with and without early postoperative complications.

Variable	No complication	Complication	p-value
Age (years)	52.0 (44.0‒58.0)	51.0 (39.0‒57.0)	0.106
Height (cm)	170.0 (165.0‒175.0)	166.5 (158.0‒173.0)	0.001
Weight (kg)	70.0 (60.0‒79.0)	61.0 (46.0‒75.0)	0.001
Albumin (g/L)	34.3 (31.0‒38.8)	33.2 (30.1‒37.2)	0.017
Creatinine (μmol/L)	76.1 (58.0‒95.0)	75.0 (48.0‒104.8)	0.350
Total bilirubin (μmol/L)	54.0 (24.7‒220.3)	118.4 (35.4‒345.4)	0.001
INR	1.4 (1.1‒1.8)	1.5 (1.3‒2.1)	0.001
Blood loss (mL)	800.0 (500.0‒1200.0)	1000.0 (500.0‒1500.0)	0.029

Data are presented as median (interquartile range). p-values were calculated using the Mann-Whitney *U*-test.

### Univariate analysis

Univariate analysis demonstrated significant differences between patients with and without postoperative complications. Body height (p < 0.001), body weight (p < 0.001), serum albumin (p = 0.017), total bilirubin (p = 0.001), INR (p = 0.001), and intraoperative blood loss (p = 0.029) were significantly associated with early postoperative complications. Among categorical variables, hepatic encephalopathy (p = 0.009) was significantly associated with postoperative complications, while ascites demonstrated borderline significance (p = 0.077). Detailed results of univariate analysis are presented in Table 2.

**Table 2:** Univariate analysis of perioperative factors associated with early postoperative complications after orthotopic liver transplantation.

Variable	Test	p-value
Age (years)	Mann-Whitney *U*	0.106
Height (cm)	Mann-Whitney *U*	<0.001
Weight (kg)	Mann-Whitney *U*	<0.001
Albumin (g/L)	Mann-Whitney *U*	0.017
Creatinine (μmol/L)	Mann-Whitney *U*	0.35
Total bilirubin (μmol/L)	Mann-Whitney *U*	<0.001
INR	Mann-Whitney *U*	0.001
Blood loss (mL)	Mann-Whitney *U*	0.029
Hepatic encephalopathy	Chi-Square	0.01
Ascites	Chi-Square	0.077

Continuous variables were analyzed using the Mann-Whitney *U*-test. Categorical variables were analyzed using the Chi-Square test. Statistical significance was defined as p < 0.05.

### Multivariable logistic regression analysis

Variables with p < 0.10 in univariate analysis were included in the multivariable logistic regression model. Correlation analysis demonstrated no evidence of severe multicollinearity among included perioperative variables (Fig. 3).

**Fig. 3:**
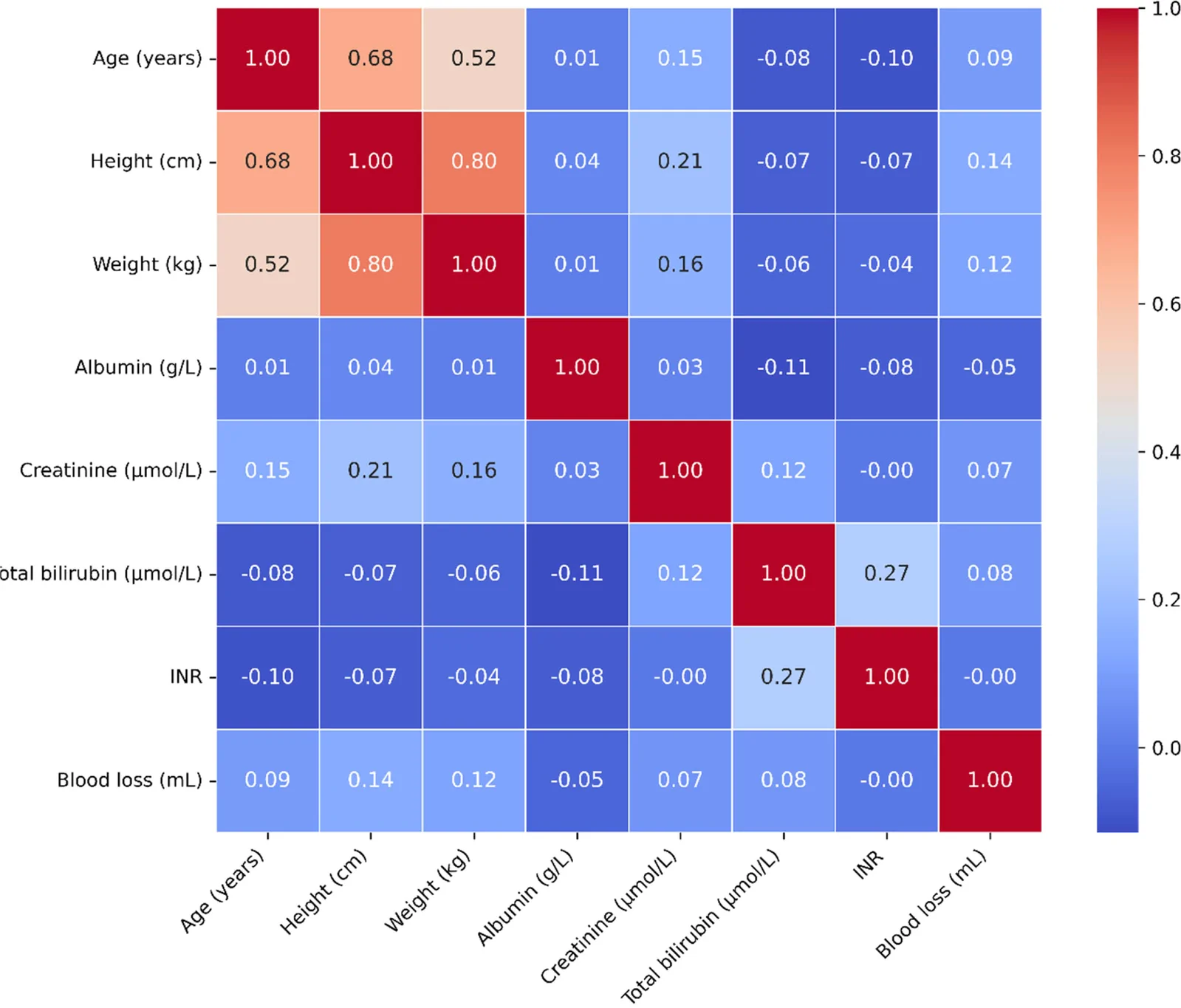
Correlation heatmap of candidate variables included in multivariable logistic regression analysis.

Hepatic encephalopathy was identified as an independent predictor of early postoperative complications following liver transplantation (OR = 1.999, 95% CI 1.23‒3.25, p = 0.005). Intraoperative blood loss demonstrated a borderline association with postoperative complications (OR = 1.00007, 95% CI: 0.99‒1.00, p = 0.052). Body weight was inversely associated with postoperative complications (OR = 0.982, 95% CI: 0.97‒0.99, p = 0.037). The complete multivariable logistic regression model is shown in Table 3. A forest plot illustrating odds ratios and 95% Confidence Intervals of variables included in the multivariable logistic regression model is presented in Fig. 4.

**Table 3:** Multivariable logistic regression analysis of predictors of early postoperative complications after orthotopic liver transplantation.

Variable	95% CI Lower	95% CI Upper	Odds Ratio	p-value
Height (cm)	0.983	1.007	0.995	0.402
Weight (kg)	0.966	0.999	0.982	0.037
Albumin (g/L)	0.947	1.01	0.978	0.179
Total bilirubin (μmol/L)	1	1.001	1	0.293
INR	0.983	1.23	1.1	0.096
Blood loss (mL)	0.999	1.014	1.007	0.052
Hepatic encephalopathy	1.23	3.251	1.999	0.005

CI, Confidence Interval; INR, International Normalized Ratio; OR, Odds Ratio. multivariable logistic regression analysis was performed using variables with p < 0.10 in univariate analysis.

**Fig. 4:**
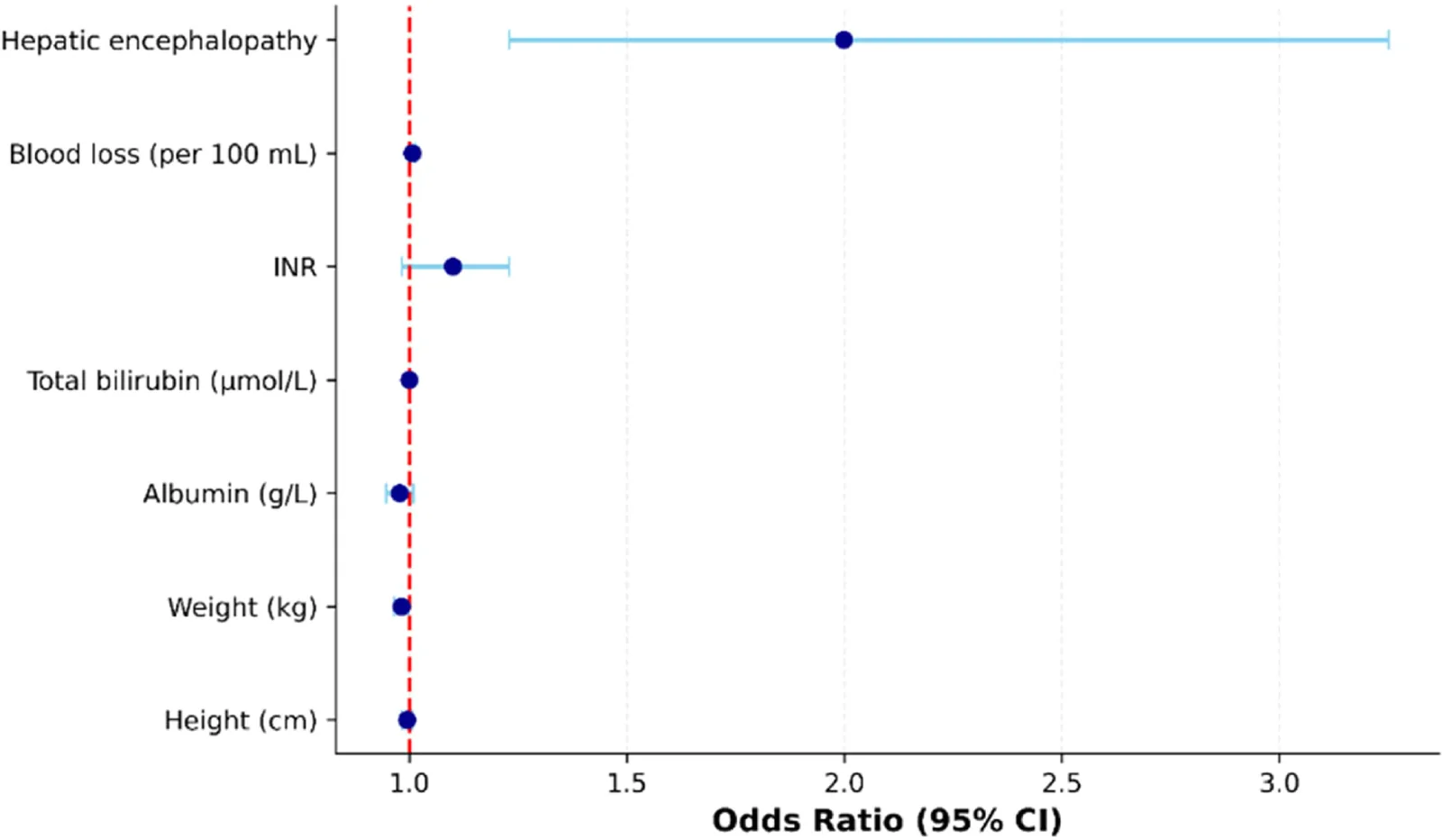
Forest plot of multivariable logistic regression analysis.

### Predictive performance of the regression model

Receiver Operating Characteristic (ROC) curve analysis was performed to evaluate the predictive performance of the final multivariable model. The model demonstrated acceptable discriminative ability with an Area Under the Curve (AUC) of 0.699. Receiver operating characteristic curve analysis demonstrated moderate predictive performance of the multivariable logistic regression model. Hosmer-Lemeshow goodness-of-fit testing demonstrated suboptimal calibration of the prediction model (χ^2^ = 21.553, p = 0.006). The ROC curve of the predictive model is shown in Fig. 5.

**Fig. 5:**
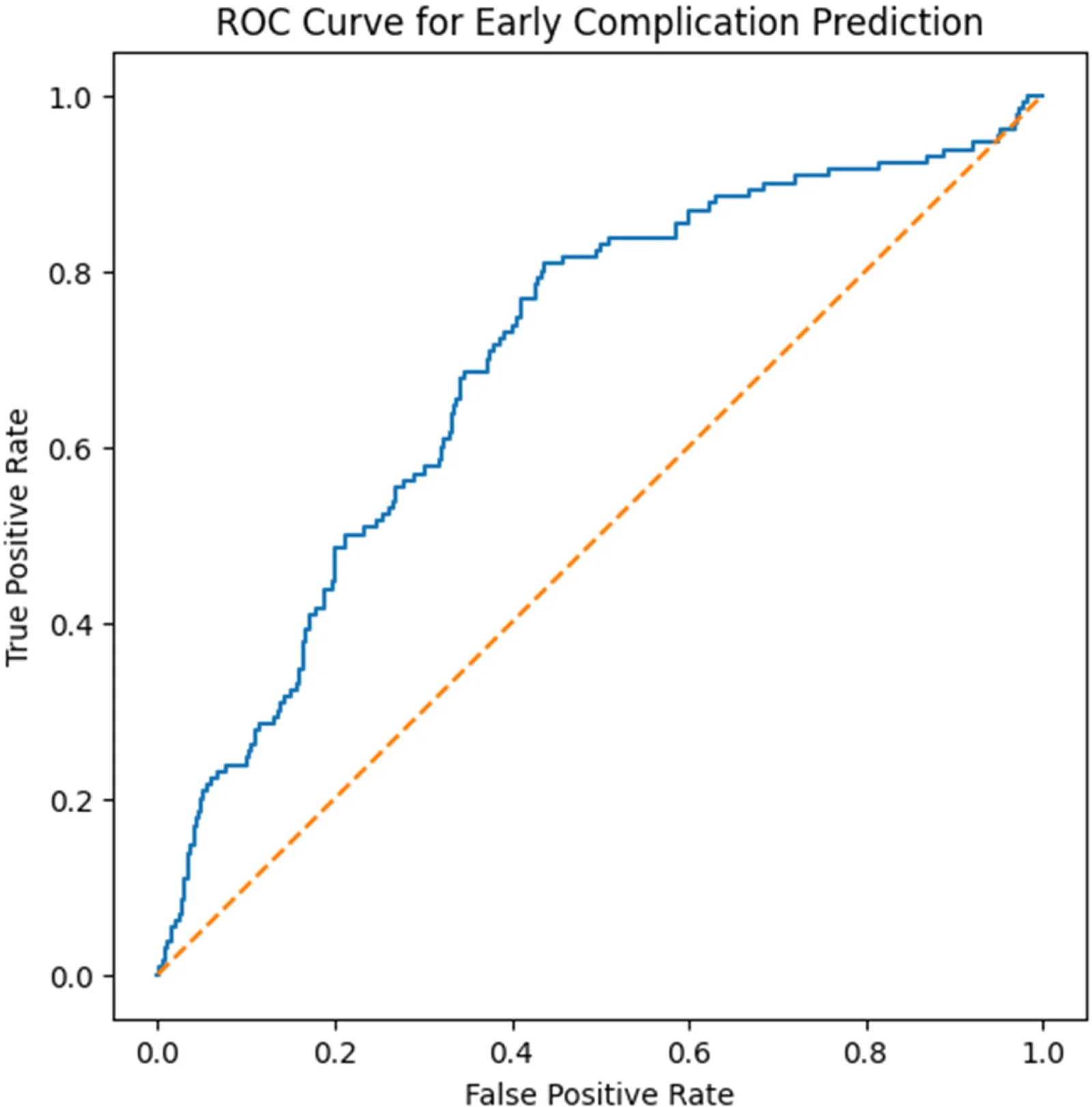
Receiver Operating Characteristic (ROC) curve of the multivariable logistic regression model for prediction of early postoperative complications.

## Discussion

The present study evaluated perioperative factors associated with early postoperative complications after orthotopic liver transplantation. The main finding of this study was that preoperative hepatic encephalopathy was independently associated with early postoperative complications following liver transplantation. Greater intraoperative blood loss may reflect increased surgical complexity and hemodynamic instability during transplantation, which could contribute to postoperative morbidity. The predictive model showed acceptable discriminative ability with an area under the ROC curve of 0.699.^13^ Hepatic encephalopathy was identified as the strongest independent predictor in the present study, increasing the odds of postoperative complications by approximately twofold. Hepatic encephalopathy is generally considered a marker of advanced hepatic dysfunction and impaired metabolic reserve. Patients with hepatic encephalopathy frequently present with severe portal hypertension, systemic inflammation, malnutrition, sarcopenia, and altered neurological status, all of which may contribute to impaired postoperative recovery after transplantation.^14^ Previous investigations have also demonstrated that pretransplant hepatic encephalopathy is associated with increased postoperative morbidity and neurological complications after liver transplantation. Neurological dysfunction and hepatic encephalopathy may negatively influence postoperative recovery in liver transplant recipients.^15^ Intraoperative blood loss demonstrated borderline statistical significance, with each 100 mL increase associated with a modest increase in postoperative complication risk. Excessive blood loss during liver transplantation may reflect technically complex surgery, severe portal hypertension, coagulation abnormalities, and hemodynamic instability. Massive intraoperative bleeding may additionally increase transfusion requirements and contribute to postoperative organ dysfunction and infectious complications.^16^ Body weight was inversely associated with postoperative complications in multi-variable analysis. Lower body weight may reflect poor nutritional status, sarcopenia, and reduced physiological reserve among patients with advanced liver disease. Malnutrition and sarcopenia are common in patients awaiting liver transplantation and have been associated with impaired postoperative outcomes and increased postoperative morbidity.^17^ The predictive performance of the final logistic regression model demonstrated moderate discrimination (AUC = 0.699). In addition, the Hosmer-Lemeshow goodness-of-fit test suggested suboptimal model calibration. Therefore, the proposed model should be considered a preliminary risk stratification tool rather than a definitive clinical prediction model. Nevertheless, the identification of preoperative hepatic encephalopathy as an independent risk factor may assist clinicians in recognizing recipients who could benefit from closer perioperative monitoring and individualized management.

Recent studies have similarly emphasized the value of perioperative risk prediction and early identification of patients at increased risk of postoperative complications following liver transplantation. Although more sophisticated prediction models and novel biomarkers continue to emerge, readily available clinical variables remain attractive because of their simplicity and immediate applicability in routine clinical practice.^18^ Future studies incorporating additional donor, recipient, and intraoperative variables, together with external validation in independent cohorts, are needed to improve model performance and clinical applicability. The composite outcome used in this study included postoperative complications with different degrees of clinical severity, ranging from relatively common conditions to serious graft-related events. Although this approach allowed evaluation of overall early postoperative morbidity, it did not distinguish the relative clinical impact of individual complications. Future studies with larger multicenter cohorts should investigate specific postoperative complications separately to determine whether their risk factors differ.

The present study has several limitations. First, this was a retrospective single-center study, which may limit the generalizability of the findings. Second, because the analysis was based on routinely collected clinical data, complete donor-related information and several important perioperative variables were not consistently available across the retrospective cohort. Donor organs originated from multiple external hospitals through the national organ allocation system, resulting in incomplete donor information for some recipients. Although some donor variables were retrieved retrospectively during manuscript revision, they could not be incorporated into the original multivariable model without reconstructing the predefined analytical dataset. Consequently, residual confounding cannot be excluded. Consequently, residual confounding cannot be excluded. Third, the composite outcome included postoperative complications with different degrees of clinical severity, which may limit interpretation of the relative contribution of individual complications. In addition, hepatic encephalopathy was evaluated as a binary clinical variable because detailed information regarding standardized diagnostic criteria, West Haven grade, and whether the episode represented a first or recurrent occurrence was not consistently available in the retrospective database. Rifaximin was not routinely used, whereas lactulose was administered only when clinically indicated. Consequently, we were unable to determine whether the severity of hepatic encephalopathy or differences in pretransplant management influenced postoperative outcomes. Future prospective multicenter studies incorporating standardized definitions and grading systems are warranted. Finally, external validation of the predictive model was not performed. Despite these limitations, the present study included a relatively large cohort of liver transplant recipients and evaluated multiple clinically relevant perioperative variables. The findings suggest that preoperative hepatic encephalopathy may serve as an important predictor of early postoperative complications following orthotopic liver transplantation.

## Conclusion

Early postoperative complications remain an important clinical problem after orthotopic liver transplantation. In the present study, preoperative hepatic encephalopathy was identified as an independent risk factor for postoperative complications, while increased intraoperative blood loss and lower body weight were also associated with poorer postoperative outcomes. These findings indicate that recipient conditions before transplantation may substantially influence early postoperative recovery. Careful perioperative evaluation and optimization of high-risk patients may contribute to improved postoperative management and reduced complication rates following liver transplantation. Further prospective multicenter studies are required to validate these findings and establish more accurate perioperative risk prediction strategies.

## Abbreviations

OLT, Orthotopic Liver Transplantation; ROC, Receiver Operating Characteristic; AUC, Area Under the Curve; CI, Confidence Interval; OR, Odds Ratio; INR, International Normalized Ratio.

## Ethics approval

This retrospective cohort study was conducted at the Organ Transplantation Center of The Affiliated Hospital of Qingdao University, Qingdao, China. Adult patients who underwent orthotopic liver transplantation between January 2014 and December 2024 were retrospectively reviewed for eligibility. The study protocol was reviewed and approved by the Medical Ethics Committee of The Affiliated Hospital of Qingdao University (Ethics Approval n°2YFYWZLL50573) and was conducted in accordance with the ethical principles of the Declaration of Helsinki.

## Consent to participate

Written informed consent was obtained from all participants or their legally authorized representatives prior to inclusion in the study.

## Authors’ contributions

Jinzhen Cai conceived and designed the study. Imran Muhammad performed data collection, statistical analysis, and data interpretation. Imran Muhammad and Faisal Ul Rehman drafted the manuscript. All authors critically revised the manuscript for important intellectual content, approved the final version, and agreed to be accountable for all aspects of the work.

## Funding

This work was supported by the National Natural Science Foundation of China (n° 82370666), the Science Foundation of Qingdao Science and Technology Bureau (n° 24-1-8-smjk-1-nsh).

## Data availability statement

The datasets generated and/or analyzed during the current study are available from the corresponding author on reasonable request.

## Declaration of competing interest

The authors declare no conflicts of interest.
