# Supplementary material for: Preoperative hepatic encephalopathy predicts early postoperative complications after orthotopic liver transplantation: a retrospective cohort study
Source: Clinics (Sao Paulo). 2026 Sep 30;81:101176. doi: 10.1016/j.clinsp.2026.101176 (PMC13654494; doi:10.1016/j.clinsp.2026.101176)
Supplement: Supplementary file 1 [file mmc1.docx]

**CLINICS-D-26-01301_Supplementary Material**

**Supplementary Table 1** Frequency of individual early postoperative complications observed after orthotopic liver transplantation.

| **Complication** | **Frequency (n)** |
| --- | --- |
| Hepatic artery thrombosis | 22 |
| Portal vein thrombosis | 26 |
| Hepatic vein thrombosis | 10 |
| Bile leakage | 16 |
| Intra-abdominal bleeding | 101 |
| Primary nonfunction | 21 |
| Delayed graft function | 4 |
| Pleural effusion | 33 |
| Edema | 0 |
| Urinary tract infection | 1 |
| Wound infection | 2 |
| Renal failure | 3 |
| Opportunistic infection | 90 |
| Cytomegalovirus infection | 0 |
